# The Pathological Aspect of Primogeniture

**Published:** 1908-04-04

**Authors:** 


### The Pathological Aspect of Primogeniture.

"The first-born child," said recently a writer in an American lay contemporary, "is practically always the best—best in brain, in build, in beauty, in everything. Why is this so? It is because married people love one another more profoundly at the beginning than afterwards; for love, like all things, grows old, grows weak, often dies." Hardly ever, of course, is it worth while to break on the scientific wheel the ephemeridæ of hasty popular journalism, but here conclusions happen to be so comically upset by facts which are left out of reckoning that a little gentle criticism may not come amiss. It is certainly true that child-study in America has revealed the association of certain favourable characteristics with early ordinal position in fraternity. Wells and others in California and elsewhere have put it on record that in scholastic proficiency, reaction time, and other simpler respects, the eldest child shows superiority. But results which, dealing with older persons, have in consequence more practical value, tell a very different story, or rather a very much more complete one. Until recently the information extant on this subject consisted mainly of the scattered *aperçus* of a few independent observers, who were often of the medical profession. Some of the findings were scarcely such as to lend themselves to idealistic popular exposition. Thus Sir A. Mitchell and also Langdon Down considered that idiots and others mentally affected were disproportionately often eldest born, while Winter said the same thing of criminals. On the other hand, Galton and Havelock Ellis described a similar characteristic, the former in eminent scientific men, and the latter in the most celebrated of those honoured by notice in the Dictionary of National Biography. Admirers of Lombroso will not fail to remark a fresh instance of the likeness between "great wits" and "madness." Havelock Ellis, in an interesting little discussion of the point, notes the diversity of characters attributed to the eldest-born child, quoting, moreover, popular sayings on the subject likewise mutually contradictory. He reconciles them by supposing that the first offspring tends mentally and physically to depart from the normal more than succeeding children, and that such departures may be in almost any direction. Quite lately some of these observations have received that expert mathematical confirmation of which statistical arguments always stand in need. Mr. Heron, of the Eugenics Laboratory, found in some valuable genealogical material of Dr. Urquhart's a markedly increased liability of the first of a family, and to a less extent of the three next eldest, to become insane. Professor Karl Pearson and Dr. Goring observed almost identically the same thing to hold good of criminals. It is probable that one may safely say as much of consumptives also. Dr. Rivers noticed in sanatorium patients an eldest-born incidence of the disease, which Professor Pearson's analysis of the material confirmed. That for all three classes the force of "diathesis"-inheritance may be greater in the case of the earlier than in that of the later members of a fraternity is the explanation provisionally suggested by Pearson. On biometrical authority, then, it may be stated that eldest children turn out criminal, consumptive, or insane oftener than do others. What might be called the "amoristic" theory of the American journalist quoted above does not, it will be remarked, apply very conveniently here. It would be sad to think that the idyllic affection of early married life had this unfortunate genetic effect. Serious students of medico-sociological matters, however, will recall that those competent observers, Korösi, the Hungarian statistician, and Marro, the criminal anthropologist, both considered that immature parents produce a degenerate stock, in which the percentage of idiots, cripples, insane, consumptives, and criminals is immensely larger than in the children of the mature. This seems to suggest a possible explanation, for, of course, eldest-born children are more likely than others to be begotten by immature persons. The whole subject is one which, in the interests both of sociology and of medicine, urgently needs investigation, the study of diatheses in particular having much to gain from the adoption of scientific and purely objective methods. Existing social conditions, however, although the introduction of anthropometrical examination of public schoolboys is a step in the right direction, are not such as to make the quest of the necessary detailed information particularly easy.

